# Dry Eye Indexes Estimated by Keratograph 5M of Systemic Lupus Erythematosus Patients without Secondary Sjögren's Syndrome Correlate with Lupus Activity

**DOI:** 10.1155/2019/8509089

**Published:** 2019-08-29

**Authors:** An Wang, Zhengyu Gu, Rongfeng Liao, Zongwen Shuai

**Affiliations:** ^1^Department of Ophthalmology, The First Affiliated Hospital of Anhui Medical University, Hefei 230022, China; ^2^Department of Rheumatology and Immunology, The First Affiliated Hospital of Anhui Medical University, Hefei 230022, China

## Abstract

**Purpose:**

To investigate the incidence, severity, and influencing factors of dry eye in systemic lupus erythematosus (SLE) patients without secondary Sjögren's syndrome (sSS).

**Methods:**

A total of 78 patients who were diagnosed with systemic lupus erythematosus and met inclusion criteria were selected as the study subjects in this cross-sectional study. Tear meniscus height (TMH) and noninvasive Keratograph tear breakup time (NIKBUT) including NIKBUT-first and NIKBUT-average of the subjects were measured using a noninvasive ocular analyzer, the Keratograph 5M (Oculus, Wetzlar, Germany). Symptoms related to dry eye were assessed using the Ocular Surface Disease Index (OSDI). The severity of SLE was evaluated by Systemic Lupus Erythematosus Disease Activity Index 2000 (SLEDAI-2K). Results of the levels of 4 serum antibodies were collected from the patients' medical records. Correlations between SLEDAI and various ocular surface parameters were analyzed, and multiple-factor binary logistic regression analysis was conducted.

**Results:**

In the study subjects, mean TMH was 0.22 mm, mean NIKBUT-first was 9.12 s, and mean OSDI was 13.14. The subjects (19 eyes) whose NIKBUT-average was < 10 s and OSDI was ≥ 13 accounted for 24.36% of all the included patients. SLEDAI showed a statistically significant correlation with TMH (*r* = −0.233, *p*=0.040), NIKBUT-first (*r* = −0.254, *p*=0.025), NIKBUT-average (*r* = −0.343, *p*=0.002), and OSDI (*r* = 0.256, *p*=0.024). According to multiple-factor binary logistic regression analysis, SLEDAI could be considered as a risk factor of the incidence of dry eye in SLE patients without sSS.

**Conclusions:**

One-fourth of the SLE patients without sSS suffered from dry eye, and the severity of dry eye correlated with the activity of SLE.

## 1. Introduction

According to Definition and Classification Report of 2017 Tear film & ocular surface society and dry eye workshop II (TFOS DEWS II) [1], dry eye is a multifactorial disease of the ocular surface. It happens when the dynamic balance of tear film is disrupted and is accompanied by ocular surface symptoms. Dry eye disease was classified into two subtypes: aqueous deficient dry eye and evaporative dry eye, in which tear film instability and hyperosmolarity, ocular surface inflammation and damage, and neurosensory abnormalities may play etiological roles. However, a recent pathophysiological study [2] supports a new scheme that aqueous deficient and evaporative dry eye may exist as a continuum. Tear deficiency is the predominant cause of immune-related dry eye, and partial or total impairment of autoimmune system could lead to reduction or even absence of tear [3].

Systemic lupus erythematosus (SLE) is an autoimmune disease that affects multiple organs in the human body [4]. The disease is characterized by erythema of cheeks, and young women are more likely to be affected. SLE patients usually experience persistent headaches, fever, swelling of joints, limited mobility, and swelling of muscle in the whole body. Meanwhile, abnormal results of laboratory tests, such as hematuria, proteinuria, low complement, low leukocyte, and low platelet, are observed in SLE patients. Accordingly, serological tests of anti-double-stranded DNA antibody (anti-dsDNA) level and the titer of antinuclear antibody (ANA) are commonly used to assess disease activity and predict lupus flare. Moreover, SLEDAI-2K questionnaire [5] is an internationally acknowledged tool to evaluate the activity of SLE. It was reported that incidence of dry eye in systemic lupus erythematosus (SLE) patients is relatively high, and thus, the pathogenesis has been explored [6–9]. It is worthy to point out that some studies [6] regarding dry eye in SLE patients ruled out the impact of secondary Sjögren's syndrome (sSS), but some [7–9] did not. Previously, a cohort study [10] has shown that 1/5 SLE patients suffered from sSS, and sSS was observed in the early course of SLE disease. Since sSS itself has nonnegligible impact on SLE patients, it is difficult to distinguish whether the damage of ocular surface is due to SLE or sSS.

To further evaluate the ocular surface condition of included SLE patients, a newly developed noninvasive technique Keratograph 5M was used in the present study. Lan et al. [11] stated that ocular surface microenvironment was very sensitive and susceptible to many factors including temperature, humidity, and sodium fluorescein. In comparison with invasive methods such as sodium fluorescein, Keratograph 5M allows assessment of ocular surface noninvasively without interfering with its balance or altering its condition [12–14]. In addition, keratograph 5M detects very early changes of tear film, displaying more sensitive detection abilities than other conventional assessment methods [15]. To the best of our knowledge, there are no studies which have investigated the ocular surface condition of SLE patients without sSS through Keratograph 5M.

The present study focused on the incidence, severity, and influencing factors of dry eye in SLE patients without sSS. The correlation between dry eye indexes and SLE severity was assessed, and multiple-factor binary logistic regression analysis was adopted to identify the risk factors of dry eye in SLE patients without sSS.

## 2. Materials and Methods

### 2.1. Participants

During February 2017 to January 2018, ophthalmic assessments such as visual acuity, slit lamp, and ophthalmoscope examination were conducted in 97 eyes of 97 participants at the First Affiliated Hospital of Anhui Medical University. For each participant, right eye was selected for measurement and statistical analysis. These subjects were diagnosed as SLE through Derivation and Validation of the Systemic Lupus International Collaborating Clinics Classification Criteria [16]. At the moment of exclusion, patients were denied of having a history of eye surgical procedures during the past year, using eye drops and contact lens in the past week. In addition, patients who suffered from cataract, retinal detachment, vitreous hemorrhage, especially pterygium, corneal scarring, and epithelium irregularity were ruled out from the research. To eliminate the impact caused by sSS, patients with positive anti-Sjögren's syndrome antigen A/Ro antibody (anti-SSA/Ro antibody) and anti-Sjögren's syndrome antigen B/La antibody (anti-SSB/La antibody) were ruled out, and the remaining 78 patients (78 eyes) denied having dry mouth symptoms. Since SLE is more common in females, 76 females and 2 male patients were enrolled in this study. The present study followed the tenets of the Declaration of Helsinki, and all patients informed consent. Moreover, the research was supported by the Clinical Ethics Committee of the First Affiliated Hospital of Anhui Medical University.

### 2.2. Dry Eye Examination

Tear meniscus height (TMH) and noninvasive Keratograph tear breakup time (NIKBUT) including NIKBUT-first and NIKBUT-average of the subjects were measured using a noninvasive ocular analyzer, the Keratograph 5M (Oculus, Wetzlar, Germany). Keratograph 5M was adjusted to fit the patient's position with the patient looking ahead; a photo was taken and particular attention was paid to the area below the pupil to record the TMH. For NIKBUT, Keratograph 5M was focused on the center of the pupil with the patient staring at the point in front of her/him. The subject was required to blink twice. Then, eyes were kept open until the subject could not tolerate. Keratograph 5M could calculate the NIKBUT-first and NIKBUT-average automatically. The average of three consecutive examination values was calculated, and the interval time was at least 60 s. Subjects were tested between 14:30 and 17:30 in a small office centrally heated to a temperature of 21°C–25°C with diffuse lighting. There were no ventilation ducts over the equipment.

### 2.3. Symptomatology Assessment

Patient symptoms were evaluated by the Ocular Surface Disease Index (OSDI) [17], a subjective questionnaire. The question asked whether the eyes had photophobia, foreign body sensation, pain, soreness, blurred vision, ghosting, and visual loss in the past week and whether eye discomfort made to suspend activities such as reading, driving at night, operating computer or bank machines, and watching TV during the past week. The scale also asked if eye discomfort occurred under the following conditions: windy weather, dry weather, and air condition. In order to quantify the symptomatology, each question had 5 levels, corresponding to different scores. The total OSDI score was then calculated on the basis of the following formula: OSDI = [(sum of scores for all questions answered) × 100]/[(total number of questions answered) × 4] [18]. Thus, the OSDI is scored on a scale of 0 to 100, with higher scores representing greater disability.

### 2.4. SLEDAI-2K

The activity of SLE was assessed by the Systemic Lupus Erythematosus Disease Activity Index 2000 (SLEDAI-2K) [5], a modification of the Systemic Lupus Erythematosus Disease Activity Index (SLEDAI) which emphasized recent skin rash and proteinuria. However, SLEDAI-2K eliminated those newly developed manifestations to focus on the continuous state of the disease. SLEDAI-2K contained 24 components, 16 of which were clinical results and 8 were laboratory results. The total score of SLEDAI-2K was the sum of all 24 descriptor scores and fell between 0 and 105. The score represented manifestations which were present at the time of the visit or in the preceding 10 days. To have a valid degree of lupus activity, a 4-level scale was used for this purpose. 0–4 points were rated as basically no activity [19], 5–9 points were defined as mild activities, 10–14 points mean moderate activities, and more than 15 points were defined as severe activities.

### 2.5. Antibody Determinations

ANA, anti-dsDNA, and anti-SSB/La antibody, anti-SSA/Ro antibody were determined from their medical records. ANA is always tested in a patient who is suspected of having SLE. Anti-dsDNA is identified to be highly specific for SLE and has strong correlation with lupus activity. It has been widely acknowledged that anti-SSA/Ro and anti-SSB/La antibody have critical roles in sSS diagnosis [20–22]. To monitor the level of all the autoantibodies, the indirect immunofluorescence method was conducted, which is based on the principle of the binding of autoantibody/antigen complexes to the immune-fluorescent secondary antibody. Afterwards, fluorescence microscope was used to observe the fluorescence representing the existence of autoantibodies.

### 2.6. Statistical Analysis

Data were expressed as mean ± standard deviation. Kolmogorov–Smirnov test was used to validate whether the data were normally distributed. Pearson linear correlation analysis was used for data that were normally distributed, and Spearman's rank correlation was applied if variables did not meet the normal distribution. The correlations were considered strong if *r* was > 0.80, moderately strong if *r* was between 0.5 and 0.8, fair within if *r* was the range of 0.3 and 0.5, and poor if *r* was < 0.30 [23]. *T*-test was introduced for normally distributed data between groups, and the Mann–Whitney test was used to assess the differences between groups if the data were not normally distributed. Chi-square test was recommended for qualitative data. The relevant parameters were taken into multiple-factor binary logistic regression model to identify the risk factors of dry eye. The significance level was set at *p* < 0.05 (both sides). Dataset and statistical analysis were performed using SPSS software 19.0 (SPSS Inc., Chicago, USA) and MATLAB software 2017b (MathWorks Inc., Natick, USA).

## 3. Results

In Table 1, the general characteristics, including age, disease duration, dry eye indexes, and serological test results, were listed. The mean value of NIKBUT-first of 78 SLE patients was 9.12 s, which was less than 10 s the normal population. Moreover, the NIKBUT-first < 5 s (25 eyes) accounted for 32.05%, 5–10 s (28 eyes) accounted for 35.90%, and >10 s (25 eyes) accounted for 32.05% of SLE patients. The NIKBUT-average < 5 s (10 eyes) accounted for 12.82%, 5–10 s (27 eyes) accounted for 34.62%, and >10 s (41 eyes) accounted for 52.56% of patients with SLE, indicating that at least 50% of the patients included in this study had abnormal NIKBUT. Although the mean value of TMH was 0.22 mm, it should be taken into consideration that the TMH of 39.74% patients (31 eyes) was less than 0.20 mm. SLE subjects had moderate ocular discomfort OSDI scores, and the mean value was 13.14.

Subsequently, the data of TMH, NIKBUT-first, NIKBUT-average, and OSDI were ranked according to the scores of SLEDAI, and the results are shown in Table 2. As the SLEDAI score increases, that is, the disease activity of SLE increases, dry eye indexes gradually change accordingly, indicating a possible correlation between the symptoms and signs of dry eye and the disease activity of SLE.

**Figure 1:**
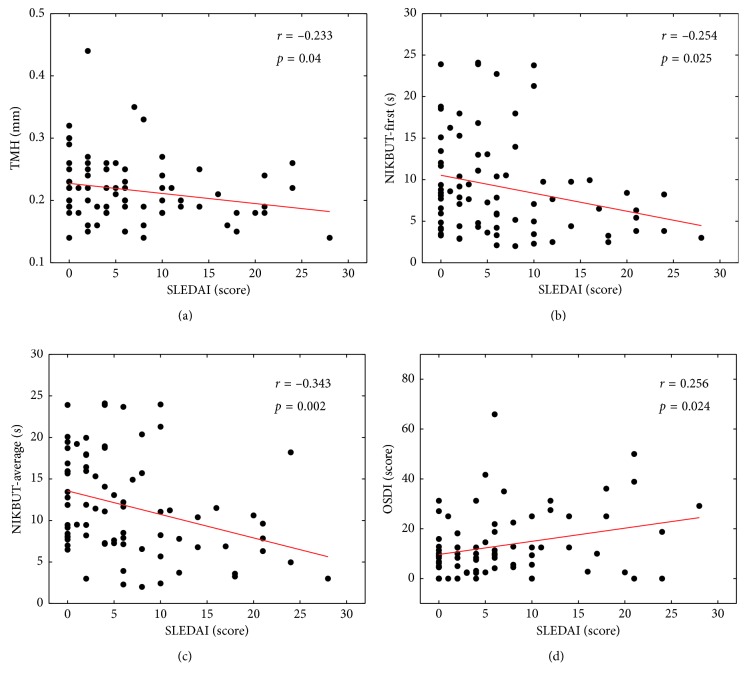
Correlations between (a) TMH and SLEDAI (*r* = −0.233, *p*=0.040), (b) NIKBUT-first and SLEDAI (*r* = −0.254, *p*=0.025), (c) NIKBUT-average and SLEDAI (*r* = −0.343, *p*=0.002), (d) OSDI and SLEDAI (*r* = 0.256, *p*=0.024) in SLE patients without sSS.

Pearson linear correlation analysis and Spearman's rank correlation coefficient analysis were performed to analyze the correlations between variables indicating lupus activity and dry eye indexes, including TMH, NIKBUT-first, NIKBUT-average, and OSDI, respectively (Table 3). The results indicated that SLEDAI were correlated with TMH (*r* = −0.233, *p*=0.040) (Figure 1(a)), NIKBUT-first (*r* = −0.254, *p*=0.025) (Figure 1(b)), NIKBUT-average (*r* = −0.343, *p*=0.002) (Figure 1(c)), and OSDI (*r* = 0.256, *p*=0.024) (Figure 1(d)). NIKBUT-first showed strong correlation with NIKBUT-average (*r* = 0.870, *p* < 0.01). No correlations were observed between ANA and dry eye parameters except the OSDI.

Based on the criteria obtained from TFOS DEWS II Diagnostic Methodology report [24], patients with OSDI ≥ 13 and NIKBUT < 10 s were enrolled into dry eye group, and the rest were defined as control group. NIKBUT-average was chosen to diagnose dry eye since its repeatability and reproducibility are better than NIKBUT-first [25]. Additionally, some other parameters were used to further explore the differences between two groups.  Table 4 showed that two groups were not significantly different in terms of age, disease duration, ANA titers, anti-dsDNA levels, and TMH (*p* > 0.05). In contrast, the scores of SLEDAI, NIKBUT-first, NIKBUT-average, and OSDI were significantly different between dry eye group and control group (*p* < 0.05), indicating incidence of dry eye was related to the severity of SLE.

Finally, multiple-factor binary logistic regression analysis was performed to examine the correlations between the incidence of dry eye with clinical characteristics and biochemical parameters, such as age, disease duration, anti-dsDNA levels, ANA titers, and SLEDAI scores in the subjects. As shown in Table 5, only SLEDAI score significantly affected the incidence of dry eye (*p*=0.007). OR value of SLEDAI score was 1.119, suggesting that SLEDAI score could be considered as a risk factor for the incidence of dry eye in SLE patients without sSS.

## 4. Discussion

Patients whose anti-SSA antibody and anti-SSB antibody were positive without any oral symptoms were ruled out in this study. Despite the fact the mean value of NIKBUT-first was 9.12 s and TMH was 0.22 mm for the SLE patients enrolled in this study, which has not met the diagnosis criteria of dry eye, it should be emphasized that NIKBUT-first of 2/3 patients and NIKBUT-average of 1/2 patients was abnormal. Moreover, almost 1/4 of the study subjects can be diagnosed as dry eye according to TFOS DEWS II Diagnostic Methodology report [24].

Statistically significant correlations were observed between the OSDI score and NIKBUT-first, NIKBUT-average, but not TMH. However, Sullivan et al. [26] suggested that no relationship could be found between any of the common signs and symptoms of dry eye disease. One explanation may be that the different clinical signs reflect different subtypes of dry eye, and each clinical sign provides distinct information regarding ocular surface conditions. Besides, the intervals between the collection of questionnaires and the examinations of signs, which were conducted a few weeks later, could also introduce errors in their study. Similarly, Kyei et al. [27] reported statistically significant associations between the OSDI scores and blink rate, contrast sensitivity scores, but not corneal staining, Schirmer test, tear breakup time, meibomian gland expressibility, and meibomian gland quality. One cause contributing to the discrepancy may be that their study subjects are first-year students who are relatively younger. Moreover, a system review [28] revealed that the correlations between dry eye signs and symptoms were between −0.4 and 0.4, indicating low-to-moderate correlation. The *r* values between dry eye signs and symptoms in this study fall into the same range.

The patients enrolled in this study were classified into dry eye group and control group according to the criteria mentioned before (Table 4). There were no differences in terms of age, disease duration, ANA titers, and anti-dsDNA levels between the two groups, indicating that the two groups were comparable. A statistically significant difference in SLEDAI scores was observed between two groups, suggesting that SLEDAI score may be related with occurrence of dry eye, and thus, the activity of SLE correlates with the incidence of dry eye.

The incidence of dry eye in SLE patients has been extensively studied. A case-control study [7] showed that tear film osmolarity in SLE group was much higher when compared with the control group. Resch et al. [8] revealed that the density of Langerhans cells in the cornea of SLE patients was greater than that in the control healthy group, supporting the idea that the increase of Langerhans cells and the change of morphology in cornea contributed to the pathophysiology of dry eye in SLE patients. Moreover, the dry eye symptoms and signs and ocular surface inflammation of SLE patients were significantly more severe than those of dry eye patients without systemic immune disease [3].

The studies about the correlation between dry eye and SLE activity in SLE patients without sSS are rare. Chen et al. [6] showed that the dry eye parameters such as corneal sensation, superficial punctuate keratopathy, and Schirmer I test exhibited moderately strong correlations (*r* > 0.8, *p* < 0.01) with anti-dsDNA level in SLE patients without sSS. Moreover, anti-dsDNA level showed high efficacy in monitoring lupus activity and that its rise predicted the relapse of SLE. The present study showed that dry eye indexes such as NIKBUT, TMH, and OSDI had correlations with SLEDAI yet at relatively low levels. One cause leading to the differences of correlations may be that this study evaluated ocular surface with noninvasive method in comparison with Chen's study. Tone et al. [29] found no correlations between the symptoms of dry eye and other objective parameters measured in children with SLE. Moreover, no differences were observed regarding Canadian Dry Eye Assessment questionnaire, tear film osmolarity, slit lamp examination, tear film breakup time, corneal fluorescein staining, Schirmer I test, and conjunctival lissamine green staining between SLE children with and without sSS group. However, the present study obtained results distinct from their studies. One possible reason may be that children have fewer symptoms compared with adults despite similar dry eye signs [30]. The other reason may be the relatively poor cooperation of children which results in measurement errors, not to mention that their study sample size is relatively small.

The present study revealed that the incidence and severity of dry eye were closely related to SLEDAI scores, suggesting the relationship between dry eye and the activity of SLE disease. However, one question remaining elusive is whether cytokine or chemokine induces dry eye in SLE patients without sSS. Lee et al. [31] elucidated that cytokines, such as IL-2, IL-4, IL-5, IL-6, IL-17, and TNF-*α* in tears, were associated with the progression of dry eye. They also found that IL-1 and IL-6 induced the proliferation of Th17 cells, which played a pivotal role in adaptive and innate immunity by releasing IL-17. Stern et al. [32] detected the relative protein level of Klk13 in serum of SS rabbit compared with control wild rabbit. They found that Klk13 appeared in SS group while absent in control group and the mRNA level of Klk13 was also upregulated in the SS group. Furthermore, they confirmed that complement played an essential role in inflammation of ocular surface. Xiao et al. [33] stated that cytokines-MMPs/MAPKs vicious cycle played pivotal roles in the development of dry eye disease. Blockage or reversal of the cytokines-MMPs/MAPKs vicious cycle relieved inflammatory responses in ocular surface tissues and alleviated damage to goblet cells, lacrimal gland, cornea, conjunctiva, etc.

ANA is deemed relevant to the severity of dry eye. Lim et al. [34] reported that SS patients with positive ANA levels (≥1 : 320) showed significantly higher conjunctival staining scores than those with negative ANA titers. Liew et al. [35] demonstrated that ANA positivity was associated with an approximately 14-fold increase in the likelihood of primary Sjögren's syndrome (pSS) versus healthy control, and the ocular surface assessed by Schirmer test, corneal fluorescein staining, conjunctival lissamine green staining, and tear breakup time was worse in patients positive in ANA. Contradictorily, the present study showed that ANA was not a risk factor influencing the occurrence of dry eye in SLE patients without sSS. One possible reason may be due to the difference of research subjects: SLE patients without sSS were enrolled in this study while their study subjects were pSS patients with relatively more severe dry eye. Another reason to explain the discrepancy might be the low specificity of ANA to reflect the disease state of SLE since the expression of ANA was relatively high in healthy individuals and ones with other autoimmune diseases [36, 37].

Based on present study, clinicians should pay attention to the ocular surface condition of patients with active SLE, and even more importantly, appropriate measures should be taken to prevent the irreversible deterioration of ocular surface. Also, if a patient with SLE history is found to have severely damaged ocular surface in his/her visits to ophthalmology department, this could be considered as an accessional indicator of SLE activity for diagnosis.

There were still some shortcomings in this study. The patients were enrolled from outpatient department of the First Affiliated Hospital of Anhui Medical University; as a result, limitations could be generated due to the area restriction and disease severity. Secondly, the subjects selected in this study were patients with SLE, a systemic disease, which requires long-term use of hormones and immune-modifiers, especially hydroxychloroquine. Yavuz et al. [38] reported that hydroxychloroquine caused damage to the ocular surface of patients with pSS. Thus, the side-effect of systemic drugs on the incidence of dry eye could not be ignored.

In conclusion, a noninvasive, newly developed technique, Keratograph 5M, was used in this study to investigate the ocular surface condition of patients with SLE yet without sSS. The results showed that SLE patients without sSS had a relatively higher risk for the incidence of dry eye, and the severity of dry eye was closely related to the disease activity of SLE. Due to the fact severe damage of ocular surface is irreversible, it is important to monitor the ocular surface of SLE patients and diagnose the disease at the early stage. Furthermore, molecular links between SLE and dry eye occurrence should be deciphered in the future.

## Figures and Tables

**Table 1:** Patient demographics.

Parameter	
Age (years)	37 ± 11
Female/male	76/2
Disease duration (years)	5.60 ± 4.32
Oral hydroxychloroquine (yes/no)	78/0
Eyedrop during the last week (yes/no)	0/78
TMH (mm)	0.22 ± 0.05
NIKBUT-first (s)	9.12 ± 5.97
NIKBUT-average (s)	11.71 ± 6.01
OSDI (score)	13.14 ± 12.92
SLEDAI (score)	6.55 ± 6.99
ANA titer	1544.36 ± 1423.68
Anti-dsDNA (positive/negative)	27/51
Anti-SSA/SSB antibody (positive/negative)	0/78

Values are expressed as average ± standard deviation. TMH = tear meniscus height. NIKBUT = noninvasive Keratograph tear breakup time. OSDI = Ocular Surface Disease Index. SLEDAI = Systemic Lupus Erythematosus Disease Activity Index. ANA = antinuclear antibody. Anti-dsDNA = anti-double-stranded DNA antibody. Anti-SSA/SSB antibody = anti-Sjögren's syndrome antigen A/B antibody.

**Table 2:** Data of TMH, NIKBUT-first, NIKBUT-average, OSDI, and ANA titer according to the severity of SLEDAI.

SLEDAI (score)	0–4 (*n* = 40)	5–9 (*n* = 16)	10–14 (*n* = 11)	≥15 (*n* = 11)
Age (years)	35 ± 10	36 ± 8	39 ± 13	42 ± 11
Duration (years)	5.07 ± 3.42	6.23 ± 5.53	7.36 ± 5.46	4.86 ± 4.04
TMH (mm)	0.23 ± 0.06	0.22 ± 0.06	0.21 ± 0.03	0.19 ± 0.04
NIKBUT-first (s)	10.43 ± 5.99	8.49 ± 5.94	8.81 ± 7.29	5.56 ± 2.52
NIKBUT-average (s)	13.76 ± 5.45	10.30 ± 6.15	10.23 ± 6.77	7.80 ± 4.53
OSDI (score)	9.04 ± 8.40	18.06 ± 16.86	14.65 ± 10.99	19.38 ± 17.68
ANA titer	1217.50 ± 1360.33	1597.50 ± 1487.17	2476.36 ± 1251.74	1723.64 ± 1443.16

Values are expressed as average ± standard deviation. SLEDAI = Systemic Lupus Erythematosus Disease Activity Index. TMH = tear meniscus height. NIKBUT = noninvasive Keratograph tear breakup time. OSDI = Ocular Surface Disease Index. ANA = antinuclear antibody.

**Table 3:** Correlation analysis between each group of data.

	Age (years)	Duration (years)	TMH (mm)	NIKBUT-first (s)	NIKBUT-average (s)	OSDI (score)	SLEDAI (score)	ANA titer
Age (years)		*r* = 0.256	*r* = −0.196	*r* = −0.263	*r* = −0.368	*r* = 0.176	*r* = 0.251	*r* = −0.017
		**p**=**0.024**	*p*=0.085	**p**=**0.020**	**p** < **0.001**	*p*=0.124	**p**=**0.027**	*p*=0.880
Duration (years)	*r* = 0.256		*r* = 0.086	*r* = 0.169	*r* = 0.125	*r* = −0.145	*r* = 0.006	*r* = −0.126
	*p*=0.024		*p*=0.452	*p*=0.138	*p*=0.275	*p*=0.206	*p*=0.955	*p*=0.272
TMH (mm)	*r* = −0.196	*r* = 0.086		*r* = 0.033	*r* = 0.174	*r* = −0.003	*r* = −0.233	*r* = 0.052
	*p*=0.085	*p*=0.452		*p*=0.771	*p*=0.128	*p*=0.980	**p**=**0.040**	*p*=0.650
NIKBUT-first (s)	*r* = −0.263	*r* = 0.169	*r* = 0.033		*r* = 0.870	*r* = −0.241	*r* = −0.254	*r* = −0.051
	*p*=0.020	*p*=0.138	*p*=0.771		**p** < **0.001**	**p**=**0.033**	**p**=**0.025**	*p*=0.660
NIKBUT-average (s)	*r* = −0.368	*r* = 0.125	*r* = 0.174	*r* = 0.870		*r* = −0.341	*r* = −0.343	*r* = −0.103
	*p* < 0.01	*p*=0.275	*p*=0.128	*p* < 0.01		**p**=**0.002**	**p**=**0.002**	*p*=0.370
OSDI (score)	*r* = 0.176	*r* = −0.145	*r* = −0.003	*r* = −0.241	*r* = −0.341		*r* = 0.256	*r* = 0.288
	*p*=0.124	*p*=0.206	*p*=0.980	*p*=0.033	*p*=0.002		**p**=**0.024**	**p**=**0.011**
SLEDAI (score)	*r* = 0.251	*r* = 0.006	*r* = −0.233	*r* = −0.254	*r* = −0.343	*r* = 0.256		*r* = 0.290
	*p*=0.027	*p*=0.955	*p*=0.040	*p*=0.025	*p*=0.002	*p*=0.024		**p**=**0.010**
ANA titer	*r* = −0.017	*r* = −0.126	*r* = −0.052	*r* = −0.051	*r* = −0.103	*r* = 0.288	*r* = 0.290	
	*p*=0.880	*p*=0.272	*p*=0.650	*p*=0.660	*p*=0.370	*p*=0.011	*p*=0.010	

TMH = tear meniscus height. NIKBUT = noninvasive Keratograph tear breakup time. OSDI = Ocular Surface Disease Index. SLEDAI = Systemic Lupus Erythematosus Disease Activity Index. ANA = antinuclear antibody.

**Table 4:** Demographic information of dry eye and control group.

	Dry eye group (*n* = 19)	Control group (*n* = 59)	Statistics	*p* value
Age (years)	41 ± 11	35 ± 10	1.903	0.061
Duration (years)	4.92 ± 4.51	5.82 ± 4.27	−0.788	0.433
SLEDAI (score)	11.37 ± 8.31	5.00 ± 5.77	−3.232	**0.001**
ANA titer	1986.32 ± 1346.83	1402.03 ± 1429.43	−1.829	0.067
Anti-dsDNA^*∗*^	6	21	0.727	0.394
TMH (mm)	0.21 ± 0.05	0.22 ± 0.05	−0.914	0.364
NIKBUT-first (s)	4.78 ± 2.21	10.51 ± 6.13	−6.065	**<0.01**
NIKBUT-average (s)	5.77 ± 2.38	13.62 ± 5.56	−8.667	**<0.01**
OSDI (score)	28.66 ± 13.50	8.14 ± 7.80	6.295	**<0.01**

^*∗*^Positive ratio of anti-dsDNA in dry eye group and control group. Values are expressed as average ± standard deviation. SLEDAI = Systemic Lupus Erythematosus Disease Activity Index. ANA = antinuclear antibody. Anti-dsDNA = anti-double-stranded DNA antibody. TMH = tear meniscus height. NIKBUT = noninvasive Keratograph tear breakup time. OSDI = Ocular Surface Disease Index.

**Table 5:** Multiple-factor binary logistic regression analysis for dry eye incidence.

Parameter	*B* value	SE value	OR value	95% CI	*p* value
Age (years)	0.046	0.029	1.047	0.989–1.109	0.115
Duration (years)	−0.090	0.074	0.914	0.790–1.058	0.227
SLEDAI (score)	0.113	0.042	1.119	1.031–1.215	**0.007**
Anti-dsDNA	−0.269	0.644	0.764	0.216–2.699	0.676
ANA titer	1.231	1.195	3.425	0.329–35.642	0.303

ANA = antinuclear antibody. Anti-dsDNA = anti-double-stranded DNA antibody. SLEDAI = Systemic Lupus Erythematosus Disease Activity Index.

## Data Availability

The primary data used to support the findings of this study can be obtained by contacting the first author through e-mail (wangan930115@163.com).
